# Diaphragmatic hernia with strangulated loop of bowel presenting after colonoscopy: case report

**DOI:** 10.1186/1755-7682-2-38

**Published:** 2009-12-11

**Authors:** Sandeep S Sodhi, Loren A Zech, Vijay Batura, Sampath Kulasekhar

**Affiliations:** 1 College of Medicine, University of Illinois at Urbana-Champaign, 190 Medical Sciences Building, 506 South Matthews Ave, Urbana, IL 61801, USA; 2 Department of Surgery, Danville Veterans Administration Hospital, 1900 East Main St, Danville, IL 61832, USA

## Abstract

**Background:**

The incidence of diaphragmatic hernias caused or exacerbated by diagnostic colonoscopy is not well elucidated at this time, and is believed to be very rare.

**Case Presentation:**

We present the case of a 57 year old man with remote history of traumatic injury who first presented with vague left shoulder pain for two weeks, mild anemia, and tested positive for fecal occult blood. Four days post colonoscopy the patient was found to have a strangulated loop of bowel herniated through the diaphragm into the left hemithorax.

**Conclusions:**

In patients with previous history of serious traumatic injury and particularly those with previous splenectomy, a thorough history and physical examination before routine colonoscopy is important. A high level of suspicion for post-operative complications should also be maintained when assessing such patients.

## Background

While data on the incidence of colonic perforation during diagnostic colonoscopy has been reported (0.045-0.65%)[1], the incidence of diaphragmatic hernias caused or exacerbated by diagnostic colonoscopy is not well defined. Case reports of transdiaphragmatic hernia first discovered during colonoscopy do exist [1-3]. One case of near fatal pneumothorax and ARDS developed after routine colonoscopy is present in the literature.[1]

It is believed that diaphragmatic injury occurs in 5% of patients with multiple traumatic injuries. Among diaphragmatic injuries resulting from blunt trauma, 70% occur on the left side primarily because the right hemidiaphragm is protected by the liver.[4] The incidence of post-traumatic diaphragmatic hernias, in general, is uncertain. Post-traumatic diaphragmatic hernias result from blunt trauma injuries such as motor vehicle accidents in approximately 80% of cases. Penetrating trauma, such as stab wounds and gunshots, are associated with the remaining 20% of cases.[4] Blunt trauma injuries are more likely to lead to herniation of abdominal contents into the thoracic cavity than are penetrating injuries primarily because the resulting diaphragmatic defect is usually larger.[4] Diaphragmatic injury may not yield herniation immediately. Over time, however, the relatively higher abdominal cavity pressure can cause protrusion of abdominal contents through even a small defect in the diaphragm and yield gradual enlargement. Interestingly, respiratory and/or abdominal symptoms following diaphragmatic hernia may manifest long after the injury, and delay in symptoms of more than ten years is not uncommon.[4] In fact, there has been report of a diaphragmatic hernia remaining asymptomatic for forty years.[5]

Here we report the case of a patient with a history of blunt trauma to the abdomen 35 years earlier who underwent colonoscopy for gastrointestinal bleeding. Four days later a strangulated loop of bowel was found at the splenic flexure contained within a diaphragmatic hernia.

## Case Presentation

A 57 year old man presented to the clinic with a chief complaint of vague left sided abdominal pain for two weeks. One year earlier he had been admitted on two separate occasions for left shoulder pain with significant tenderness over the bicipital groove, lateral border of the scapula, and sternocleidomastoid. The patient had survived a mine explosion in 1967 while serving in the Vietnam War resulting in surgical asplenia. Physical examination at the time of presentation was unchanged from previous admissions. CBC revealed a decreased RBC count (4.47 cells/L) with a borderline low hemoglobin level (14.5 gm/dL) and a slightly elevated MCV (98.1 fL). Fecal occult blood study was positive.

A colonoscopy was performed under IV sedation with 25 mg of meperidine and 2 mg of midazolam. Colonoscopy revealed a large number of diverticula in the sigmoid and descending colon. The scope was negotiated up to the splenic flexure at which point the lumen was no longer visualized. The patient was turned supine and to the right lateral decubitus position. Despite these maneuvers the colonoscope could not be advanced past the splenic flexure. The scope was withdrawn and the injected air was removed.

**Figure 1:**
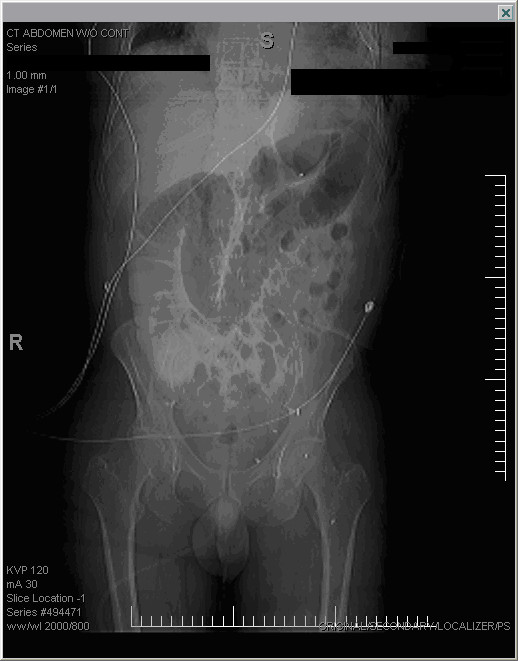
**Upright CT abdomen without contrast obtained 24 hours after colonoscopy**. Upright view shows herniation of large bowel through left hemidiaphragm and into thoracic cavity.

**Figure 2:**
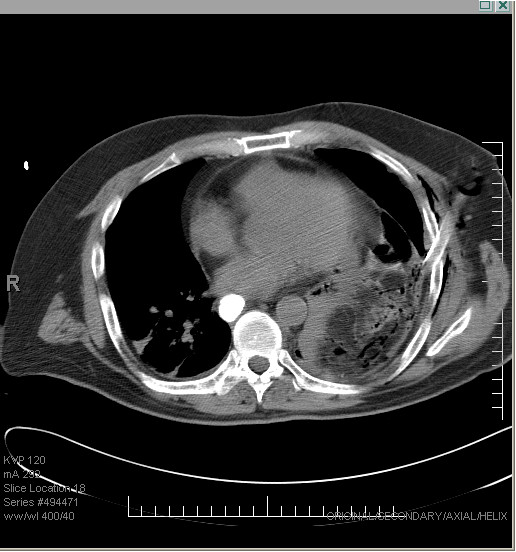
**Abdominal CT scan after placement of chest tube**. Reveals presence of loops of bowel in thoracic cavity as well as fluid in left hemithorax. Note also mediastinal shift to the right side.

Within 24 hours of colonoscopy the patient presented to the emergency department with complaints of abdominal distension, epigastric pain, nausea, and dyspnea. A CT scan of the abdomen was interpreted as normal. The patient was discharged with analgesics. Two days later he returned with similar complaints. A second CT scan was read as normal. The patient was again discharged. Four days post colonoscopy the patient was admitted to the hospital due to worsening pain and dyspnea. He had not had passed flatus or stool since the colonoscopy. Physical examination now revealed a tachycardic, diaphoretic man in respiratory distress with a distended abdomen and faint bowel sounds. Laboratory analysis revealed the following at the time of admission: urine positive for protein, WBC's, and RBC's; serum WBC 11.3 cells/L; RBC 4.12 cells/L; Hgb 13.1 gm/dL; HCT 39.4%; MCV 96.6 fL; PLT 306 K; neutrophils 51.3%; monocytes 9.9%; glucose 140 mg/dL; BUN 54 mg/dL; Cr 4.4 mg/dL; Na 133 mEq/L; K 3.6 mEq/L; Cl 97 mEq/L; CO2 17 mEq/L; Ca 8 mEq/L; cardiac enzymes CK 213 U/L; CK-MB 0 U/L; troponins 0 U/L; Mb 124.9 ng/ml; pH 7.42; pCO2 24 mmHg; pO2 71 mmHg; HCO3 15.6 mmol/L; O2-sat 78% on room air, 98% on 4 L. Abdominal imaging revealed distended right and transverse segments of the colon without gas in the left side of the colon or free air under the diaphragm (Figure 1). Chest x-ray showed infiltrates in the left lower lung. A CT scan revealed a large amount of air and fluid in the left hemithorax causing a shift of the mediastinum to the right. Soft tissue density was also noted on the left side of the diaphragm (Figure 2). A chest tube was placed on the left side and 800 ml of foul smelling fluid was drained and sent for culture. Follow-up imaging revealed that the lung field had reexpanded to 75% and the patient's shortness of breath had improved. Analysis of the pleural fluid revealed gram positive cocci in pairs - later shown to be enterococcus and an alpha strep species.

The patient was treated for sepsis. He was given a 1 L bolus of lactated ringer's solution and maintained on D5 in normal saline at 200 ml/hour. Acute renal failure secondary to sepsis led to a metabolic acidosis. A CT scan of the abdomen without contrast revealed herniation of the stomach and splenic flexure of the colon through the left diaphragm with extensive inflammatory changes in the area of the splenic flexure. Marked thickening of the colonic wall suggested incarceration. Exploratory laparotomy revealed that the splenic flexure had herniated through a defect in the posterolateral portion of the left diaphragm. Six inches of strangulated bowel was removed with adjoining mesentery. The defect in the diaphragm was closed and a cecostomy was created. The patient was treated with vancomycin and gentamicin.

The patient developed hypoxia soon after leaving the operating room. An EKG revealed ST segment elevation in the lateral leads. Seven days after admission the patient expired.

## Conclusions

It is important to consider the potential causes of the transdiaphrgmatic hernia encountered in this patient. The primary issue is whether a new hernia was caused by routine colonoscopy or an existing defect was exacerbated. Given this patient's history of trauma to the region from a mine explosion and his resultant splenectomy it is likely that a defect did exist prior to colonoscopy. Moreover, it is plausible that the patient's history of left sided shoulder pain and his original presenting complaint of left sided abdominal pain one year earlier were due to diaphragmatic injury and herniation. Diagnostic colonoscopy revealed a narrowed lumen that was impassable. Insufflation likely worsened the existing diaphragmatic defect and bowel herniation.

It is vital that, when possible, a patient's entire medical history be carefully reviewed prior to proceeding with medical intervention. It is known that small diaphragmatic defects or those protected by the liver may prevent herniation of abdominal contents into the chest and may remain asymptomatic for months to years.[5] In fact, strangulation of abdominal viscera may provide the first evidence that a diaphragmatic hernia is present.[5]

In managing patients with existing or suspected diaphragmatic hernias careful examination of a chest radiograph is important. However, this modality is diagnostic in only 50% of the cases.[4] The use of rapid helical computed tomography with sagittal reconstruction facilitates diagnosis and should be given serious consideration when there are post-operative complaints.

## Consent

Reasonable attempts were made to contact next of kin for publication of this case report and any accompanying images. This case presentation was reviewed by the appropriate committees and administrators at both the Danville Veterans Administration Hospital and the College of Medicine at UIUC.

## Competing interests

The authors declare that they have no competing interests.

## Authors' contributions

SSS reviewed the patient history and wrote the first draft of this case report. LAZ edited subsequent drafts of the manuscript and created the abstract. VB performed the diagnostic colonoscopy on the patient and reviewed the manuscript. SK arranged for digitized radiographic images and edited the manuscript. All authors read and approved the final manuscript.
